# “Just another fish in the pond”: the transitional care experience of a hip fracture patient

**DOI:** 10.5334/ijic.1103

**Published:** 2013-06-26

**Authors:** Justine Toscan, Brooke Manderson, Selena M Santi, Paul Stolee

**Affiliations:** School of Public Health and Health Systems, University of Waterloo, Ontario, Canada; School of Public Health and Health Systems, University of Waterloo, Ontario, Canada; School of Public Health and Health Systems, University of Waterloo, Ontario, Canada; School of Public Health and Health Systems, University of Waterloo, Ontario, Canada

**Keywords:** transitional care, hip fracture, ethnography, qualitative research, patient experience, caregivers

## Abstract

**Introduction:**

Miscommunication and lack of coordination can compromise care quality and patient safety during transitions in care, especially for medically complex older adults. Little research has been done to investigate care transitions from the perspective of those receiving and providing care.

**Methods:**

This study explored multiple care transitions for an elderly hip fracture patient, post-surgery. Interviews and observations were conducted with the patient, their family caregivers, and health care providers, at each point of transition between four different care settings.

**Results:**

Four key themes were identified over the patients care trajectory: ‘Missing Crucial Coversations’—Patient and family caregivers did not feel involved or informed about decisions in care; ‘Who’s Who’—Confusion about the role of health care providers; ‘Ready or Not’—Not knowing what to expect or what is expected; and, ‘Playing by the Rules’—Health system policies and procedures hinder individualized care.

**Conclusion:**

Study findings point to the need for the health care system to engage patients and family caregivers more fully and consistently in the process of care transitions as well as the importance of understanding these processes from multiple perspectives. Recommendations for system integration are proposed with a focus on transitional care.

## Introduction

Successful care transitions are critical for the effective and efficient treatment of older persons with multiple chronic conditions. Care transitions are defined as periods of time when individuals are moved to new care settings according to changes in their health and functional status [1]. Typical transitional care settings for older adults in Canada include, but are not limited to, acute hospital care, in-patient rehabilitation, complex continuing care, convalescent care, home care, and long-term care [2–5]. When older adults with complex medical issues experience a care transition, multiple health professionals from different disciplines are involved across various settings, expanding the patient’s circle of care [6]. It is during these types of complex transitions, where quality of care and patient safety have been found to be at greatest risk [7–13]. Outcomes of poor transitional care include medication errors [14], increased use of hospital, ambulatory, and emergency services [15, 16] as well as poor client satisfaction [17].

Studies of transitional care to date have typically focused on a single point of transition between care settings [18]. A focus on one transitional experience fragments the patient’s journey into a ‘snap-shot’ of their experience, rather than examining their experiences over the entire care trajectory [19]. Further, these studies tend to emphasize the health care provider perspective of transitional care, rather than all key players involved [18]. In particular, there is limited documentation and understanding in the literature of transitional care from the perspective of patients and their family caregivers.

Older persons with hip fracture are a complex, frail population who frequently present with multiple co-morbid conditions [20], and who generally receive care from multiple providers across a variety of settings [21]. Surgical orthopaedic patients are an example of a population that frequently experiences multiple care transitions across the continuum. Specifically, hip fracture is an archetypal geriatric health issue and the most common injury requiring hospitalization among older adults [22]. On average, hip fracture patients transition to 3.5 care settings before reaching their final discharge destination [23].

Additional information on relevant care pathways in Ontario are described by Alzahrani and colleagues [24], to guide planning around surgery, length of stay, and hospital discharge for hip fracture patients. The pathway recommends that hip fracture patients be operated on within 48 hours of their fracture and transferred to an inpatient rehabilitation unit or facility 5 days post-surgery, if medically stable. A seven to 10-day assessment and plan exists to help establish whether patients can return to the community after a 28-day inpatient rehabilitation stay or should transition to complex continuing care or specialized geriatric services [24]. Patients discharged from in-patient rehabilitation to the community receive a home care assessment to determine the level of home care services required through the Community Care Access Centre [24]. With such a complex care trajectory, hip fracture patients represent an ideal population in which to examine transitional care experiences from multiple perspectives.

The purpose of this study was to explore the experience of transitional care over the complete care trajectory for a single hip fracture patient, from multiple perspectives, including the patient, family caregivers, and relevant health care providers. This study was conducted as part of a larger Canadian program of research—‘InfoRehab’ (www.inforehab.uwaterloo.ca)—a multi-site ethnographic study that is focused on care transitions for older adults with musculoskeletal disorders [3–5, 25, 26].

## Methods

### Approach/Design

Care transitions are largely experiential and subjective events rather than defined clinical points in a patient’s care trajectory (i.e. admission and discharge), and as such, they are difficult to measure or quantify [5]. Accordingly, researchers required a qualitative, reflexive approach that places emphasis on context in order to study the experiences of transitional care from multiple key perspectives [27].

This study employed a focused ethnographic approach. As the researchers had ongoing and frequent interactions with study participants across the complete care trajectory, an interpretivist epistemological stance was applied to this inquiry to control for (but not eliminate) bias [28]. An interpretive lens allowed researchers to gain an understanding of patient experiences of transitional care, using both verbal and non-verbal cues, and relied on their own experiential knowledge as context for interpreting the data [29]. Researchers worked alongside participants in their respective care environments to collect interview and observational data as a patient transitioned between various care settings [30].

Self-reflexivity, the process of understanding how the research process affects the researchers and vice-versa [28, 30–32], was ongoing throughout both the collection and data analysis stage. Data were collected using three main processes of investigation: current literature, semi-structured interviews, and participant observation [28]. Triangulation was achieved through understanding transitional care from three different perspectives including that of the patient, family caregiver, and health care provider across multiple settings, which together formed the complete patient experience [30].

Ethics clearance for the study was received from the University of Waterloo, Office of Research Ethics and the relevant hospital(s)/facilities research ethics boards.

### Sampling and participant characteristics

Sampling for this study was purposive [33]; pre-established study criteria included being a hip fracture patient who was expected to undergo multiple transitions in care (i.e. at least one transition or more), with involved family caregivers, and multiple providers, over the age of 65 years and proficient in written and spoken English. This ensured that participants could be fully involved in the interview process. The only exclusion criterion for this study was a diagnosis of moderate to severe cognitive impairment, since participants were asked to retrospectively recall events from their experiences in previous care settings.

The hip fracture patient (referred to herein as Mrs. Smith), was recruited from a mid-sized city in Southern Ontario, Canada through a non-academic large community-based hospital with just under 500 beds. An acute care resource nurse assisted in the recruitment of Mrs. Smith, as she made initial contact with potential research participants prior to contact from the researchers. Recruitment of Mrs. Smith led to an inclusive recruitment process for members of her circle of care, including family caregivers and health care providers at each point of transition between care settings. Informed consent from Mrs. Smith allowed researchers to approach family caregivers and health care providers involved in her care to inquire if they would be willing to participate in the interview process. Discharge to an affiliated in-patient rehabilitation hospital with complex continuing care occurred following acute care. The patient then transferred to an assisted living facility where she convalesced. The final discharge destination was to her home where the patient was assessed through the local Community Care Access Centre and received home care services and supports through a local home care provider agency through a brokerage model of care [34]. In total, the perspectives of Mrs. Smith, two of her family members, and ten health care providers were incorporated in this study.

Mrs. Smith’s general demographic characteristics (e.g. age, sex, comorbidities) were typical of a hip fracture patient [35]. A retired nurse in her 80s, Mrs. Smith was a widow who lived alone in an apartment complex. She initially fractured her left hip and underwent surgery and recovery in an acute care setting, and then subsequently fractured her right hip from a fall that occurred on the day of hospital discharge.

### Data collection

Data were collected by trained members of the research team [JT, BM] [28]. Face-to-face semi-structured interviews were conducted to achieve an in-depth, rich description of each participant’s experience of transitional care. Mrs. Smith, her family caregivers and health care providers were asked to openly describe their experiences at each point of transition. Interviews were conducted using semi-structured interview guides [36]. All three types of interviews (patient, family caregivers, and health care providers) led to in-depth conversations with participants over a 30 to 60 minute time period [37].

Researchers used various techniques to accurately reflect content and circumstances of the interviews. All interviews were recorded and transcribed verbatim. Observational notes were recorded in the form of a memo to document individuals’ non-verbal cues, such as body language and facial expressions, as well as the environmental setting of the interviews [33]. These data were then organized and analyzed to understand the complexities of Mrs. Smith’s overall transitional care experience.

**Figure 1.**
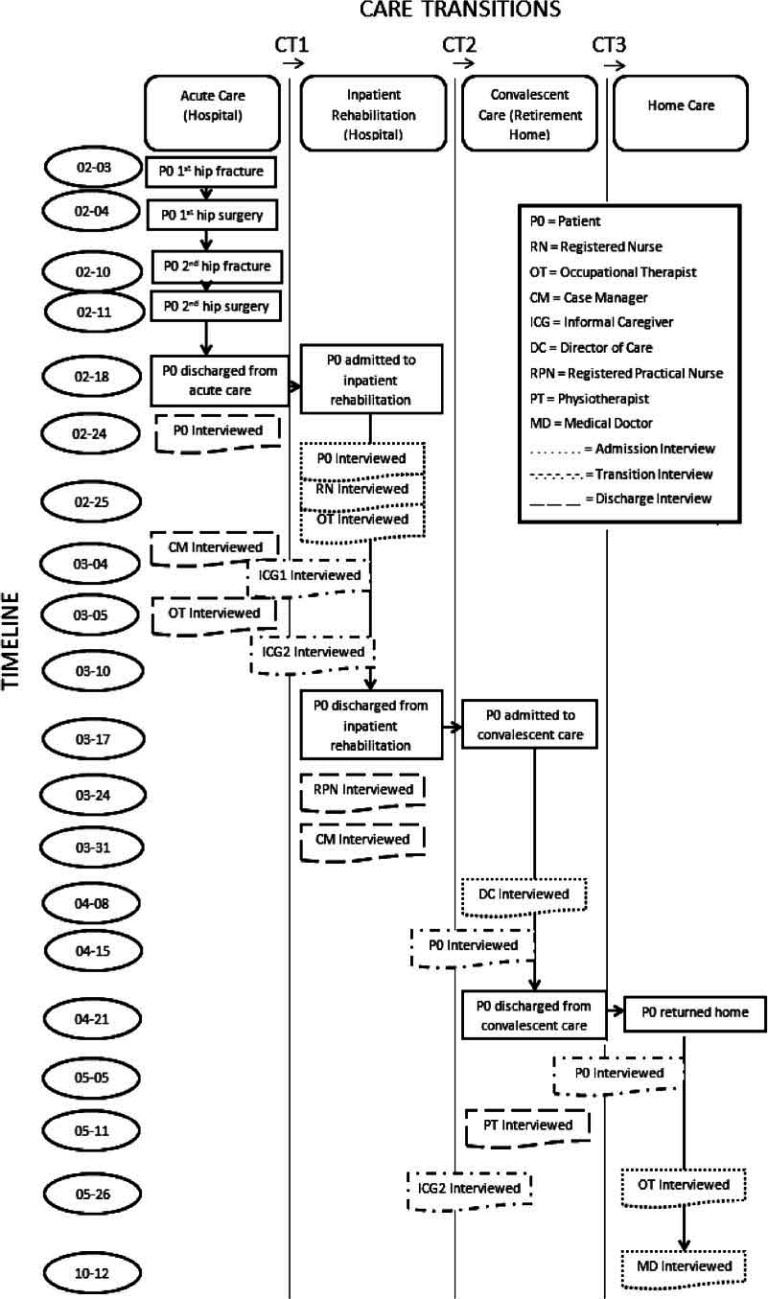
Map of care transition interviews with participants over time.

Figure 1 is a visual depiction of the research interviews that took place at each point of transition for Mrs. Smith, who was interviewed, and the timeline for each transition and interview [38]. Mrs. Smith received care in four different settings before returning home. In total, sixteen interviews were completed, involving thirteen participants.

### Data analysis

The analysis followed an inductive approach; researchers allowed the themes to emerge directly from the data [39], guided by three linked processes: data reduction, data display, and conclusion drawing [40]. Data reduction began with the analysts [JT and BM] coming together several times to discuss how to approach the data. To stay true to the pre-determined interpretivist epistemological stance, analysts decided to approach the data through the multiple lenses of each of the participants in order to develop an understanding of the overall patient experience of transitional care [33].

To ensure findings were authentic representations of the multiple perspectives included in the study, both analysts became immersed in the data and individually reviewed each of the transcripts and observational notes for key themes. Short snippets of data, called ‘meaning units’, that were relevant to the overall patient experience were individually abstracted from the transcripts. Related ‘meaning units’, within and across the different perspectives were then grouped and condensed (where possible) to eliminate redundancies. Observational data were used to attach importance and environmental context to the ‘meaning units’ as they were identified and condensed (see Table 1).

Data display included further condensing the ‘meaning units’ into categories to describe common threads across interviews and perspectives and develop a thick description of Mrs. Smith’s overall transitional care experience. At this stage in the analysis, researchers met to bring together their separate initial categories and worked with the larger research team to come to agreement on one overarching framework of themes derived from the data. Conclusion drawing brought meaning and analysts went back to the data set to verify essential thematic structures and to capture any previously unrecognized relevant ‘meaning units’ under each category [40].

## Results

**Figure 2.**
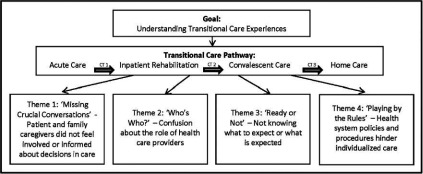
Emerging qualitative themes.

Four themes emerged from the multi-perspective data to describe the transitional care experience of Mrs. Smith across her entire care trajectory. The overarching trend across all themes was that care transitions present multiple challenges not only for patients, but also for family caregivers and health care providers. These challenges were grouped into four themes and reflect similarities across all three perspectives, but are also explained in relation to each participant’s specific role in transitional care (see Figure 2).

### Theme 1: ‘Missing Crucial Conversations’—Patient and family caregivers did not feel involved or informed about decisions in care

One theme evident in the data was that Mrs. Smith and her family caregivers felt unimportant and left out of the decision-making process surrounding their own, or their family member’s care plan. Both Mrs. Smith and her family caregivers expressed their concerns during interviews with being left out of crucial conversations and decisions around care. Specifically, Mrs. Smith described her frustrations with having little input in terms of her next steps in care:

“They gave me a slip yesterday saying you’re going to be discharged on [date]. That’s it you know. The hell with you, whether you want to or not or whether you feel you’re capable. Now I know they can’t leave it up to the person themselves because some of them would stay in here forever. But I think there should be some communication between the person that’s involved and maybe say, “What do you think about going at such and such a time? Do you feel that you’re making enough progress?” And I guess, not that I’m patting myself on the back, but I think I would be smart enough to give them the right answer…”

Mrs. Smith went on to further describe her experience with not being treated as a participant in her own health care, or given the opportunity to direct decision-making processes by using an analogy to compare her situation to that of a helpless fish:

“There’s more to health than just physical health. There’s that feeling of support, of not being a—well just another fish in the pond. I guess that’s the way I’m feeling here. You’re just another fish in the pond. And when they come along with the hook they’ll pull you up and if you’re trout they’ll put you one place, and if you’re…they’ll put you another place, and if you’re pike, they’ll put you another place.”

Mrs. Smith’s family caregivers echoed their mother’s concerns about not feeling informed and updated about important elements of care. For example, one daughter described her experience when health care providers failed to notify her about her mother’s second hip fracture. This cost one of her daughters a lengthy and unnecessary trip to the hospital under the impression that Mrs. Smith would be discharged that day. Mrs. Smith’s family members described their additional challenges managing communications outside of the immediate circle of care, and especially when there are issues with effective communication within the circle of care. According to one of Mrs. Smith’s daughters, family tension can result from poor information exchange between formal health care providers and family caregivers:

“It’s challenging enough when you’ve got three adults in three different locations with busy lives. It’s challenging enough to keep the information flowing when it flows smoothly, but when it’s not kind of flowing then it really becomes challenging and can create some family tension.”

### Theme 2: ‘Who’s who’—Confusion about the role of health care providers

Another theme that arose was lack of clarity in health care provider roles. This lack of clarity tends to exacerbate already fragmented care responsibilities during transitions between settings and providers. Within disciplines, role confusion was discussed as an issue largely due to the constant and quick turnover of staff across a variety of care settings. Specifically, several health care providers described a lack of consistency in staff shift changes making it difficult to keep track of their duties for each individual patient. For example, one nurse discussed the difficulty she encountered in determining the amount of orientation the patient received from previous nursing staff upon admission to the inpatient rehabilitation unit:

“There is a pamphlet but I personally didn’t—something about rehab pamphlet. I don’t know. I don’t know if she even received it, I just remember hearing them talk about it. I did not give it to her because I’m still looking for this myself… Yeah, it should have been [done by another nurse]. I don’t know if it was done.”

Health care providers indicated that an over-reliance of informal channels of communication such as conversations and telephone calls seems to promote redundancies in the information communicated between care team members. Additionally, role confusion between disciplines was also identified, where there is a lack of knowledge regarding health provider roles outside of their own scope of practice. For example, between Community Care Access Centres and hospitals, nurses often have different levels of understanding regarding the role of case managers. For example, one case manager stated:

“Um, just if the nurses… I would like to think know our role. Some of them know it a lot better than others…it makes a difference for the information I give the client.”

Finally, while role clarity was evidently frustrating for health care providers themselves, both within and between care disciplines, it was also apparent based on interview and observation data, that Mrs. Smith and her family caregivers both had difficulty distinguishing between members of the formal health care team. Mrs. Smith had difficulty physically recognizing health care providers due to unmarked uniforms, and understanding who to direct questions to. Mrs. Smith also expressed her concerns in directing her questions to meet her care needs:

“It’s not that I don’t remember, I wouldn’t know [who to ask] anyway because you don’t know whether they’re a nurse, health provider or whether they’re just one of the people that serve the meals. You don’t know…”

### Theme 3: ‘Ready or not’—Not knowing what to expect or what is expected

Another theme that emerged from the data was that Mrs. Smith and her family caregivers did not feel equipped or prepared for the care transitions as they occurred. All those who were interviewed involved in the care transition process for Mrs. Smith expressed feeling unprepared and overwhelmed with their individual role in care. For example, Mrs. Smith described the rushed nature of her transition between acute care and inpatient rehabilitation, and not being told the differences between the two care settings:

“… you leave acute care and boom, you’ve got something else. There’s no preparation for the patient. There’s no communication between those. So you come from one when you’ve gotten this, you go to another the next day where you don’t get that, but you do have to do this, you don’t have to do that. It’s just overwhelming.”

The acute care setting and inpatient rehabilitation setting were described and observed to be quite different in terms of patient expectations; with inpatient rehabilitation requiring patients to take a more active and participatory role in their care. However, Mrs. Smith was unable to recognize this change in expectations until well after she arrived to a new setting, and expressed that she would have appreciated being aware of differences between settings prior to her transition.

Both family caregivers attributed their mother’s anxiety to the rushed nature of the transition process:

“I just think it was pretty unsettling for my mom. To just sort of be yanked out of the hospital and taken to [inpatient rehabilitation] hospital.”

“…she was really quite anxious about this meeting she was going to have about her discharge from (rehabilitation hospital) because it was really fast. I think she figured she’d be in (rehabilitation hospital) like probably close to a month and all of a sudden after a week they had a target date for her that was only a couple of days, you know. So I think she’s been a little bit anxious and I mean she is 87, like it makes a lot of difference, you know. The older you get the harder it is to make these decisions and the more anxiety comes along with them.”

Additionally, quick care transitions were described by a nurse as creating heighted anxiety for Mrs. Smith, limiting her ability to absorb information:

“Even the next day I had to reiterate where to go because it was her first day, and she was overwhelmed about being put in a new hospital. Of course, someone’s bound to forget where to go, and this place is situated in circles. So I find myself walking in circles all the time. And so, I just had to retell her where [the dining room] was.”

### Theme 4: ‘Playing by the rules’—Health system policies and procedures hinder individualized care

The final theme that emerged was that health system policies and procedures restrict health care providers’ ability to deliver individualized care. Throughout the transition process, Mrs. Smith, her family caregivers and health care providers mentioned various policies and procedures, which hindered the delivery of quality care. One major component of high quality care that was recognized during interviews was health care providers’ ability to tailor approaches to individual needs. According to health care providers, the health care system’s focus on discharge-centred care planning contributed to the pressure to initiate transitions with Mrs. Smith, and others, before they felt comfortable doing so. An occupational therapist working within an inpatient rehabilitation hospital pointed out:

“I think, I mean there are always areas to improve and I think one of the biggest problems right now that we’re facing is that there is pressure to have people discharged quickly, and there may not be always services available for them when they go home. And a lot of time we would like to keep people here longer than we do.”

This issue resonated across the three perspectives and presents an important safety concern for older patients transitioning to care settings with fewer services. Another policy challenge that was described by health care providers was feeling restricted by admission criteria set by each care facility, requiring patients to need a certain level of care to meet eligibility criteria. Mrs. Smith only qualified for two facilities in the community, and these limited options were described by one of her daughters as creating heightened anxiety:

“She didn’t meet the criteria for [convalescent care] so [that] wasn’t an option based on the PT and OTs [Physical Therapists and Occupational Therapists]. And then, so we say ‘Ok… can’t go here.’ I think she thought [long-term care home] potentially…and at that time [long-term care home] had a prohibitive waitlist, like three weeks or so. And she was too good for [long-term care home] as well. You need to have a certain element of need for care rather than just uh… a want.”

The uncertainty of transitional care is heavily influenced by strict eligibility policies at long-term care homes and their attempts to match these with the constantly fluctuating functional levels of frail older adults. As well, privacy tools in care facilities were described as a communication barrier between family caregivers and health care providers. Specifically, one of Mrs. Smith’s daughter communicated that she was unable to receive any information regarding her mother’s status upon transitioning to a new setting, causing great concern:

“I phoned [the care setting] that day…to find out how she was doing and they wouldn’t tell me anything because I didn’t have her privacy code…I talked to the switchboard and I said you know, I’m really desperate to find out how my mom’s doing.”

## Discussion

For patient care to benefit fully from multiple providers working together within and across settings, the health care system needs to engage patients and family members more fully and consistently in care and decision-making processes [41]. There is also a need to support more effective communication between disciplines and across care settings—extending involvement within the patient’s circle of care and reinforcing accountability during transitions [2, 42, 43]. The results of this study are consistent with the literature that examines the intricacies of transitional care, especially for medically complex older adults [2, 17, 44, 45]. The inclusion of multiple perspectives across one patient’s care trajectory adds to the current literature by reinforcing the challenges, complexities, and confusion around transitional care.

Suter and colleagues [46] describe “10 key principles for successful health systems integration”. Relevant principles of integration for transitional care according to data collected in this study include: 1) having a patient focus; 2) standardized care delivery through interprofessional teams; 3) governance structures; and 4) organizational culture and leadership [46]. Each of the resulting study themes points to a breakdown in one or more areas of health system integration.

The first theme, “patient and family caregivers did not feel involved or informed about decisions in their care”*,* describes the risk of a patient losing their sense of confidence and control around decision-making and involvement in their care. Inclusivity of patients and their families in this process therefore means creating opportunities for them to share their opinions, feelings, and insights while engaging and participating in their care [41, 46]. Involvement in care promotes confidence and control, and fosters a sense of engagement in the management of conditions and care [47]. While being patient focused can be done through a joint care planning session, other techniques include the development of care contracts [48]. As described by Toscan [48], developing care contracts with family caregivers as soon as patients are admitted to acute care (post-hip fracture surgery) is one possible strategy to support shared decision-making, as it allows for the definition of roles and for clearly establishing expectations and responsibilities at the onset of care for all parties involved [49], and prepares them for their role in providing care [41]. It also recognizes the contributions of caregivers throughout the transitional care process [41].

The second theme, “confusion about the role of health care providers”, describes the issue of role confusion due to a constant and quick turnover of staff across multiple settings. This makes it difficult to decipher health care providers’ roles and responsibilities. As described by Suter and colleagues [46], this issue corresponds to “standardized care delivery through interprofessional teams.” Clearer roles and responsibilities lead to greater care continuity and smoother transitions across settings [5, 46]. The use of best practice guidelines, care pathways, and decision-making tools can help to integrate the care provided across the continuum. Other outcomes include issues around helping the patient and their families to distinguish between formal care providers and their respective roles in the patients’ circle of care. Clarifying care responsibilities, reducing redundancies, and lessening confusion has the potential to minimize fragmented care and promote continuity within and across settings [41, 46].

The third theme, “not knowing what to expect or what is expected”, describes how participants felt unprepared and overwhelmed with their individual role in care. This speaks not only to the need for a greater patient focus and standardized care delivery through interprofessional care teams, but also to governance structures that promote care coordination across settings and levels of care [46]. Assumptions that patients and families will automatically adapt to different environments, especially in medical crisis situations, are unrealistic in working towards a goal integrated transitional care. Both patients and family caregivers need to be prepared for care transitions and changes in settings, which are both structurally and functionally different [50]. This builds an understanding about site specific care provision and expectations for the patient as well as the role of the family in supporting the patient [41]. Preparing patients prior to transitioning to a new care environment includes a number of recommendations such as providing them with an orientation package including a tour of the new site and a written description of what they can expect their stay to look like. Having a care provider that is known to the patient [49] attend an orientation session allows for greater continuity of care and for questions to be addressed in advance of the transition.

The final theme, “health system policies and procedures hinder individualized care”, recognizes that certain policies and procedures may restrict patient care and hinder the patient-provider relationship that they were initially intended to serve. These include models such as discharge-centred care planning, privacy and protection laws around the sharing of patient information, and restricted admission criteria [46, 49]. To promote a patient-centred approach, health care providers need to have flexibility and freedom to use their professional judgment to make decisions through the use of appropriate tools [4, 46, 49]. This is linked to improving information systems that allow for greater informed care and exchange of information as well as the standardization of care delivery [46]. Overall, proper training, regular circulation of patient updates, and education around the use of electronic tools may be necessary to ensure efficient and effective use of patient information among a variety of health professionals and to begin to understand population-level needs and outcomes [51]. A navigator or guide to assist individuals who are new to the caregiving role and/or setting may also alleviate stressors and provide the necessary information and training to support patients and families [49, 52].

This study possesses several strengths. First, due to the complexity of medical care required by Mrs. Smith, she was a memorable patient for all participants involved, which enhanced the depth and richness of the interviews conducted. Her background as a nurse reduced issues with health literacy, and aided the flow and ease of conversations. While Mrs. Smith’s transitional care experiences highlight the complexities of moving between providers and settings, experiences of others with less health system knowledge and insight may have been more challenging. The fact that the patient had two consecutive hip fractures from the time she was admitted to acute care until discharge also speaks to the depth of her experience and involvement with acute care and a variety of health care providers.

It should be noted that, although very detailed descriptions of transitional care from multiple perspectives were elicited, some of the experiences may be unique to the individual patient, especially considering differences in background, experience, care trajectories, settings, and providers. In particular, our results may not generalize to hip fracture patients without support from family caregivers. Also, while hip fractures represent an archetypal complex geriatric illness and thus provide an opportunity for rich insights into transitional care experiences, the generalizability of these results to other complex patients should be further explored in future research.

## Conclusion

This study reflects on the challenges with family caregiver and health care provider involvement experienced by older adults as they transition through the health care system. By gaining a rich understanding of Mrs. Smith’s experiences gathered through multiple perspectives, this research points to areas for improvement that will lead to greater system integration. Because of the complex health problems and challenges experienced by a hip fracture patient, this patient’s experience provides a rich opportunity to identify many of the health system challenges that would be experienced by other older patients with multiple morbidities. Our study has also given a voice to a patient as a partner in care as well as being a recipient of care. Through the inclusion of multiple perspectives the importance of both transformative care and patient experiences for quality improvement can be realized [53]. As stated by the National Health Services Confederation [53],

“Improving the experiences of all patients starts by treating each of them individually to ensure they receive the right care, at the right time, in the right way for them” [p. 3].

## Figures and Tables

**Table 1.** Coding chart for theme 1—‘Missing Crucial Conversations’.

Quote	Meaning unit	Condensed meaning unit	Category
“They gave me a slip yesterday saying you’re going to be discharged on [date]. That’s it you know. The hell with you, whether you want to or not or whether you feel you’re capable. Now I know they can’t leave it up to the person themselves because some of them would stay in here forever. But I think there should be some communication between the person that’s involved and maybe say, “What do you think about going at such and such a time? Do you feel that you’re making enough progress?” And I guess, not that I’m patting myself on the back, but I think I would be smart enough to give them the right answer…”	“But I think there should be some communication between the person that’s involved and maybe say, “What do you think about going at such and such a time? Do you feel that you’re making enough progress?”	Communication between patient and health care providers	Lack of communication
			
“There’s more to health than just physical health. There’s that feeling of support, of not being a—well just another fish in the pond. I guess that’s the way I’m feeling here. You’re just another fish in the pond. And when they come along with the hook they’ll pull you up and if you’re trout they’ll put you one place, and if you’re…they’ll put you another place, and if you’re pike, they’ll put you another place.”	“I guess that’s the way I’m feeling here. You’re just another fish in the pond. And when they come along with the hook they’ll pull you up and if you’re trout they’ll put you one place, and if you’re…they’ll put you another place, and if you’re pike, they’ll put you another place.”	Feeling like just another fish in the pond	Lack of autonomy
			
“It’s challenging enough when you’ve got three adults in three different locations with busy lives. It’s challenging enough to keep the information flowing when it flows smoothly, but when it’s not kind of flowing then it really becomes challenging and can create some family tension.”	It’s challenging enough to keep the information flowing when it flows smoothly, but when it’s not kind of flowing then it really becomes challenging and can create some family tension.”	Challenging to keep information flowing	Feeling uninformed
